# NASSJ commentary: patient travel burden and postoperative outcomes after lumbar fusion

**DOI:** 10.1016/j.xnsj.2026.100948

**Published:** 2026-09-23

**Authors:** Ashraf N. Nawari, Jonathan N. Grauer

**Affiliations:** Department of Orthopedics and Rehabilitation, Yale School of Medicine, 47 College St, New Haven, CT 06510, United States

## Commentary

Meng et al. performed a study entitled “Patient travel burden and postoperative outcomes after lumbar fusion” that was published in the *North American Spine Society Journal* [1]. This study utilized an institutional database to assess the correlations between travel distance / driving time to the hospital and postoperative length of stay, readmissions, medical adverse events, and surgical complications.

The authors of the discussed study hypothesized that increased travel time and distance to the treating hospital might be independent risk factors for adverse outcomes. They identified adult patients who underwent lumbar fusion for degenerative or traumatic pathology at their academic center between January 1, 2013, and November 15, 2025. Patient variables were abstracted, as well as 90-day medical complications and 1-year surgical complications.

A total of 403 patients met inclusion criteria. The mean age at surgery was 51.42 ± 10.66 years, with an average body mass index (BMI) of 30.51 ± 5.89. The average travel distance to the hospital was 44.35 ± 47.94 miles, and the average estimated travel time was 60.16 ± 50.09 minutes. In multivariable logistic regression, shorter travel time was only associated with a greater likelihood of nonhome discharge (OR 0.9941, p = .04). Travel time and distance to hospital were not associated with any other postoperative adverse outcomes (p ≥ .05 for all).

The overarching finding in the study was a lack of significance with travel burden across almost all postoperative outcomes. The marginally significant association identified between travel burden and nonhome discharge disposition (OR 0.9941, p = .04) was interpreted as not being of major clinical significance and potentially related to factors that could not be completely controlled.

As the authors note, these findings support the concept that travel to highly specialized centers may be feasible without clearly contributing to adverse postoperative outcomes. However, it is also noted that patients who travel longer distances may represent a particularly motivated and resourced group with the ability to overcome geographic barriers and comply with follow-up. Although the authors attempted to control for variables such as social vulnerability index (SVI) that could account for this effect, unmeasured patient-level characteristics may still influence the observed null relationship between travel burden and postoperative outcomes.

Nonetheless, the reviewed study contributes to the literature by suggesting that travel burden should not be assumed to contribute to worse postoperative outcomes after lumbar fusion. The lack of significant adverse associations with travel time/distance is reassuring but it does not eliminate the need to identify patients for whom travel remains a barrier. Future prospective studies may evaluate socioeconomic resources, the presence of home health support, and patient-reported outcomes to better delineate those who electively travel for specialized care from those for whom distance may be a factor.

## Declaration of competing interests

The authors declare the following financial interests/personal relationships which may be considered as potential competing interests: Co-author is Editor-in-Chief of North American Spine Society Journal, and Deputy Editor of Journal of the American Academy of Orthopaedic Surgeons—J.G. Given his role, J.G. had no involvement in the peer review of this article and had no access to information regarding its peer review. Full responsibility for the editorial process for this article was delegated to another journal editor. If there are other authors, they declare that they have no known competing financial interests or personal relationships that could have appeared to influence the work reported in this paper.
